# Enhancing endoscopic measurement: validating a quantitative method for polyp size and location estimation in upper gastrointestinal endoscopy

**DOI:** 10.1007/s00464-024-10758-2

**Published:** 2024-03-11

**Authors:** Nazanin Safavian, Simon K. C. Toh, Martino Pani, Raymond Lee

**Affiliations:** 1 Faculty of Technology, University of Portsmouth, Portsmouth, UK; 2 Department of Upper GI Surgery, Queen Alexandra Hospital, Portsmouth Hospital University NHS Trust, Portsmouth, UK

**Keywords:** Endoscopy, Polyp, Size estimation, Electromagnetic tracking system

## Abstract

**Background:**

Accurate measurement of polyps size is crucial in predicting malignancy, planning relevant intervention strategies and surveillance schedules. Endoscopists’ visual estimations can lack precision. This study builds on our prior research, with the aim to evaluate a recently developed quantitative method to measure the polyp size and location accurately during a simulated endoscopy session.

**Methods:**

The quantitative method merges information about endoscopic positions obtained from an electromagnetic tracking sensor, with corresponding points on the images of the segmented polyp border. This yields real-scale 3D coordinates of the border of the polyp. By utilising the sensor, positions of any anatomical landmarks are attainable, enabling the estimation of a polyp’s location relative to them. To verify the method’s reliability and accuracy, simulated endoscopies were conducted in pig stomachs, where polyps were artificially created and assessed in a test–retest manner. The polyp measurements were subsequently compared against clipper measurements.

**Results:**

The average size of the fifteen polyps evaluated was approximately 12 ± 4.3 mm, ranging from 5 to 20 mm. The test–retest reliability, measured by the Intraclass Correlation Coefficient (ICC) for polyp size estimation, demonstrated an absolute agreement of 0.991 (95% CI 0.973–0.997, *p* < 0.05). Bland & Altman analysis revealed a mean estimation difference of − 0.17 mm (− 2.03%) for polyp size and, a mean difference of − 0.4 mm (− 0.21%) for polyp location. Both differences were statistically non-significant (*p* > 0.05). When comparing the proposed method with calliper measurements, the Bland & Altman plots showed 95% of size estimation differences between − 1.4 and 1.8 mm (− 13 to 17.4%) which was not significant (*p* > 0.05).

**Conclusions:**

The proposed method of measurements of polyp size and location was found to be highly accurate, offering great potential for clinical implementation to improve polyp assessment. This level of performance represents a notable improvement over visual estimation technique used in clinical practice.

Endoscopy is crucial for diagnosing gastrointestinal diseases and detecting early malignancies. In the management of gastrointestinal diseases, optical assessment during endoscopy is essential for following strategies like “diagnose and leave” and “resect and discard”, as well as setting surveillance intervals and choosing resection techniques. Enhancing this assessment can optimise patient care, balance intervention risks and benefits, and manage healthcare resources effectively. One important factor in predicting the malignancy risk and growth rate is the size of detected lesions, such as polyps. Symptomatic hyperplastic polyps larger than 10 mm with pedunculated morphology should be resected [1]. Polypectomy of fundic gland polyps of 10 mm or larger, and hyperplastic polyps of 5 mm or larger is recommended [2]. The endoscopic mucosal resection (EMR) technique for gastric lesions smaller than 10 mm in size and the endoscopic submucosal dissection (ESD) technique for lesions larger than 10 mm in size are recommended [1]. Larger gastric polyps require more frequent surveillance [3]. Diminutive colorectal polyps less than 5 mm are targeted for “diagnose and leave”, or “resect and discard” strategies [4].

In current clinical practice, both endoscopists and pathologists estimate polyp size. Studies show discrepancies regarding whether measurements by pathologists should influence clinical decisions [5–7] or those by endoscopists [8], with a majority favouring histopathological measurements. The visual estimation made by endoscopists in vivo is challenging because the size of an object, as viewed in the image of the monitor, changes depending on the distance between the endoscope camera and the object. Therefore, guessing the size visually is subject to error and variability. On the other hand, histopathological measurements are not applicable in strategies such as “diagnose and leave” or “resect and discard”. Moreover, factors such as resecting the rim of the normal tissue, and shrinkage after polypectomy can manipulate the actual in vivo size. Misjudging the polyp size, either by overestimation or underestimation [9, 10], may result in inaccurate decisions regarding the necessity for resection or surveillance [7, 11]. Consequently, the advised follow-up schedule may not correlate with the actual risk level, leading to either unnecessary, frequent surveillance or insufficient monitoring. Inaccurate polyp size estimation may also influence the selected resection technique, potentially reducing the rate of complete and curative resection [12]. All these factors impact patient outcomes and healthcare system costs.

To overcome this challenge, several studies have proposed different approaches for in vivo polyp measurement, including the use of an endoscopic graduated device [13–15], applying deep learning models [16, 17], and integrating specialised devices with conventional endoscopes [18–22]. Among these, the integration of a specialised device seems to hold significant promise for providing a direct, objective, and accurate measurement.

In our prior study [23], we developed a novel quantitative method to provide polyp size and location measurements based on integrating an electromagnetic tracking sensor with a conventional endoscope evaluated in an upper gastrointestinal experimental model. Building on our previous work, the main aim of this study is to further investigate the quantitative method applicability by conducting ex vivo experiments to assess the system’s performance under conditions more resembling real endoscopy procedures.

## Materials and methods

The authors declare that no human subjects were involved in this study, and as such, IRB approval was not required.

### System overview

**Fig. 1:**
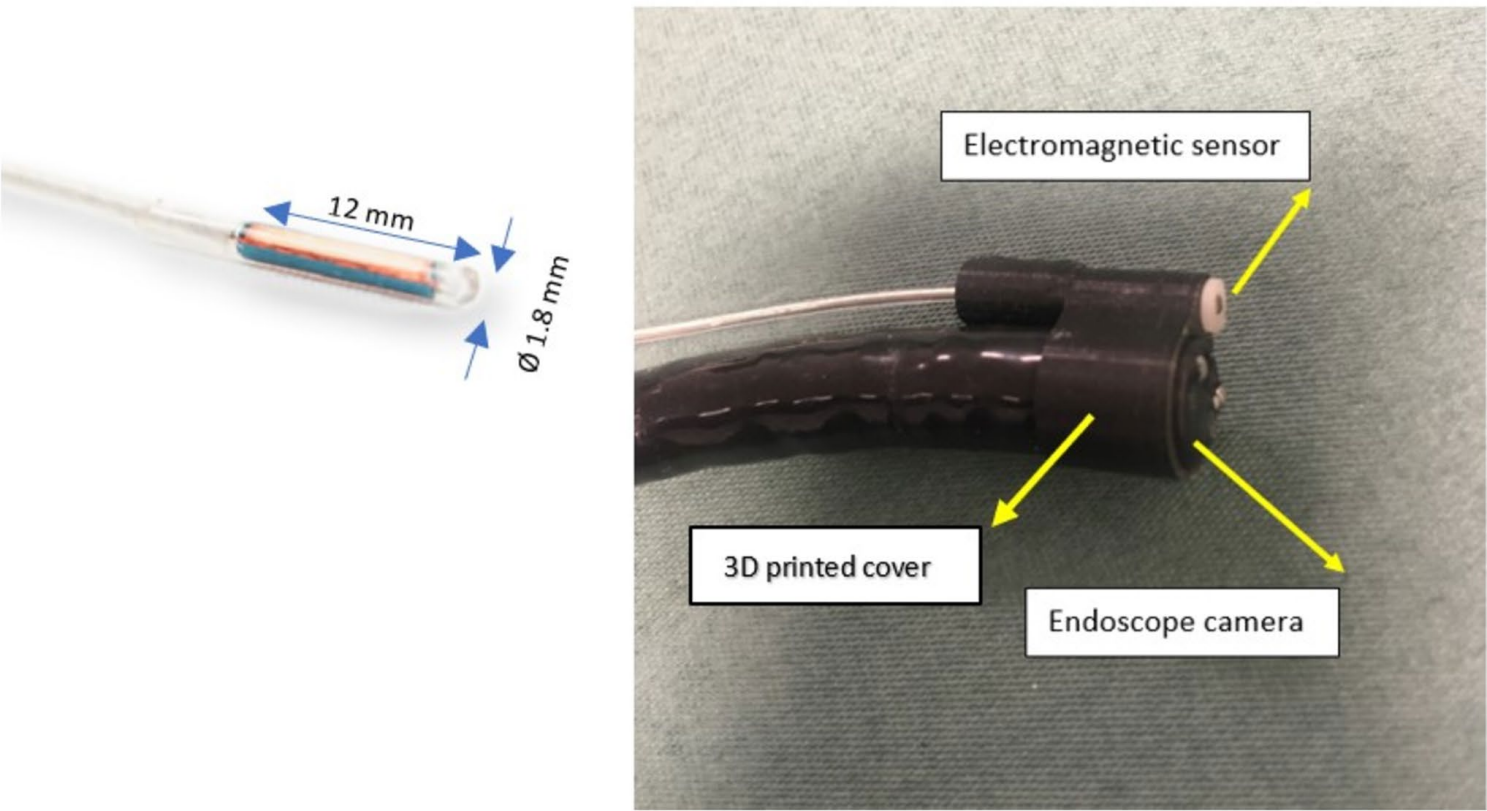
Right: Endoscope with a fixed electromagnetic sensor using a 3D printed cover. Left: Dimensions of the electromagnetic sensor

In our previous research, we introduced a novel quantitative method, which consists of integrating electromagnetic tracking technology^1^ with a conventional endoscope (Pentax EPK-i), as illustrated in Fig. 1, and is complemented by a newly developed computer-vision-based algorithm. This algorithm can yield the 3D coordinates of a detected polyp’s border in real scale. By fitting an ellipse to these 3D point coordinates, one can derive both the longest length of the polyp and the distance from its centre to a specific anatomical landmark, as detailed in our previous work [23]. Evaluated in a simulated upper gastrointestinal model and an artificial rounded polyp, a root mean square error (RMSE) of less than 1 mm for size and 3 mm for location estimation were achieved.

The sensor accuracy test results indicated no significant degradation in sensor performance in endoscopic settings, underscoring its potential applicability. Moreover, through sensitivity analysis, we provided recommendations for capturing endoscopic images from a polyp to ensure optimal accuracy with our proposed method. This includes avoiding large relative movements (specifically, relative translation > 30 mm or relative rotation > 30 degrees), minimal movements (displacement < 3 mm), or pure forward–backward movements.

Following the promising initial results, the quantitative method was evaluated in this study, while endoscopy procedure was performed using ex vivo pig stomachs. While the core principles of the quantitative method remain intact for this evaluation, a few modifications were necessary to adapt to the unique characteristics of the new setting. These modifications related to the two steps of the computer vision-based algorithm including finding corresponding points and polyp segmentation.

While the movement of the polyp cannot be controlled, signs of its movement might be detected when multiple image pairs are used for a single polyp size determination. Therefore, here a simple approach of capturing multiple images and subsequently multiple estimations to compensate for potential polyp movements between taking image pairs were investigated. If the polyp remains stationary, the 3D coordinates of the polyp’s centre would be expected to stay consistent across all measurements. Consequently, outliers in the estimated 3D coordinates of the polyp centres were identified using quartiles, and those image pairs were excluded from the final estimation, while the average of remaining estimations was considered as final estimation.

In the following sections first, two adapted steps including polyp segmentation and corresponding points will be described briefly, and subsequently, the experimental design for method evaluation will be explained.

### Polyp segmentation

**Fig. 2:**
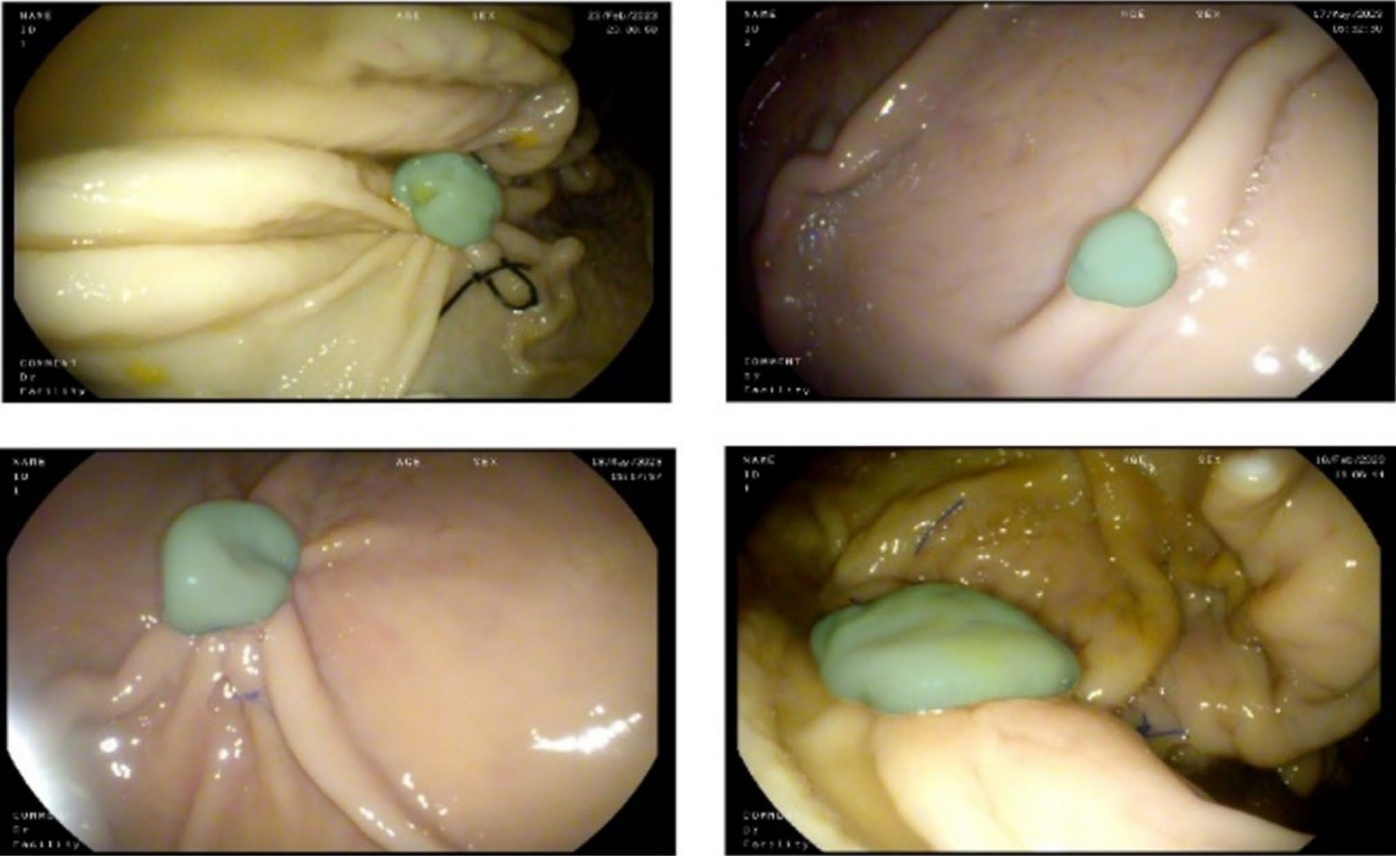
Visualisation of ex vivo polyp images. Green: polyp segmentation results using Plop-PVT model followed by post-processing operations (Color figure online)

Given the diverse characteristics of polyps, which reflect the variability observed in actual polyps, here we employed a pre trained DL-based model called Polyp-PVT (Pyramid Vision Transformers) [24], for polyp segmentation purposes in our experiment. This model consists of a pyramid vision transformer [25] as an encoder to extract multi-scale feature maps where the image divided to smaller patches in each level. The model consists of three other modules, a cascaded fusion module (CFM) to fuse the high-level features through progressive integration, a camouflage identification module (CIM) to capture low-level features using an attention mechanism, and a similarity aggregation module (SAM) to integrate low and high-level features for final segmentation. The Polyp-PVT model aims to extract more powerful and robust features to improve polyp segmentation regarding accuracy and generalisation ability. The model achieved a Dice similarity coefficient (DSC) of 0.917 and 0.937 on the Kvasir-SEG [26] and CVC-ClinicDB [27] that consist of 1000 polyp images and 612 polyp frames, respectively. For our ex vivo experiment, we employed this model and subsequently applied some image post-processing techniques to further improve the segmentation quality including, basic morphological operations such as opening, and filling operations, to eliminate small detected regions of interest (ROI), and fill in small holes within the ROIs, or deformable segmentation based on active contour models [28] in situations where the model output covers the polyp partially. Figure 2 illustrates some examples of segmentation outputs on polyp images as explained above.

### Corresponding points

Repetitive and indistinctive texture surfaces in endoscopic images can adversely affect the feature detection and matching algorithms. Consequently, in this paper, we explore a new approach for determining corresponding points based on shape context, which is unaffected by the texture-less nature of the endoscopic images.

**Fig. 3:**
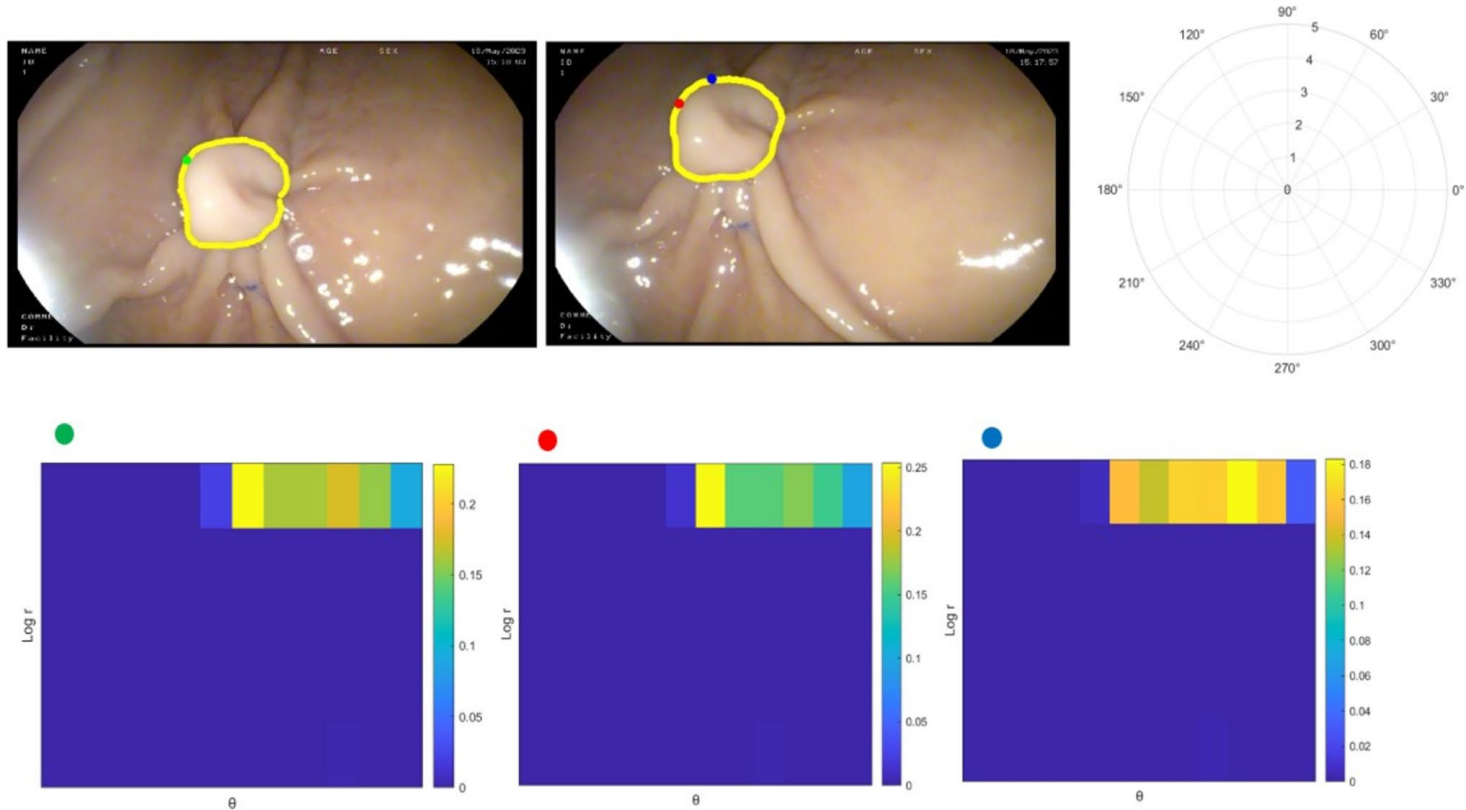
Concept of the Shape Context. Top row: Sample points on the border of one polyps in two endoscopic images and the diagram of a log-polar coordinate system with five bins for log r and 12 bins for ɵ. Bottom row: Shape context for reference samples marked by green, red and blue, on the polyp border (Color figure online)

An object’s shape can be represented by a discrete set of points, denoted *P* = ${p}_{1}$, ${p}_{2}$, …, ${p}_{n}$ where ${p}_{i}$ ∈ R2 from the internal or external contours of the object. In the case of a polyp, the shape can be defined by a set of points located on its border. Given the set of points P, with n points of the polyp shape in one image and the set of points Q, with m points in the other image, the shape context method [29] can be used to find one-to-one correspondences between these two 2D sets of points, P and Q. The shape context is a vector of local geometric information that characterises the distribution of points around a reference point on the shape boundary. The shape context can be defined as a histogram using a log-polar coordinate system. The log-polar system represents a point based on the logarithm of its distance from the origin (log r) and its angle (θ) from a reference direction. This is illustrated in Fig. 3 with a log-polar coordinate system of 5 bins for log r and 12 bins for θ.

**Fig. 4:**
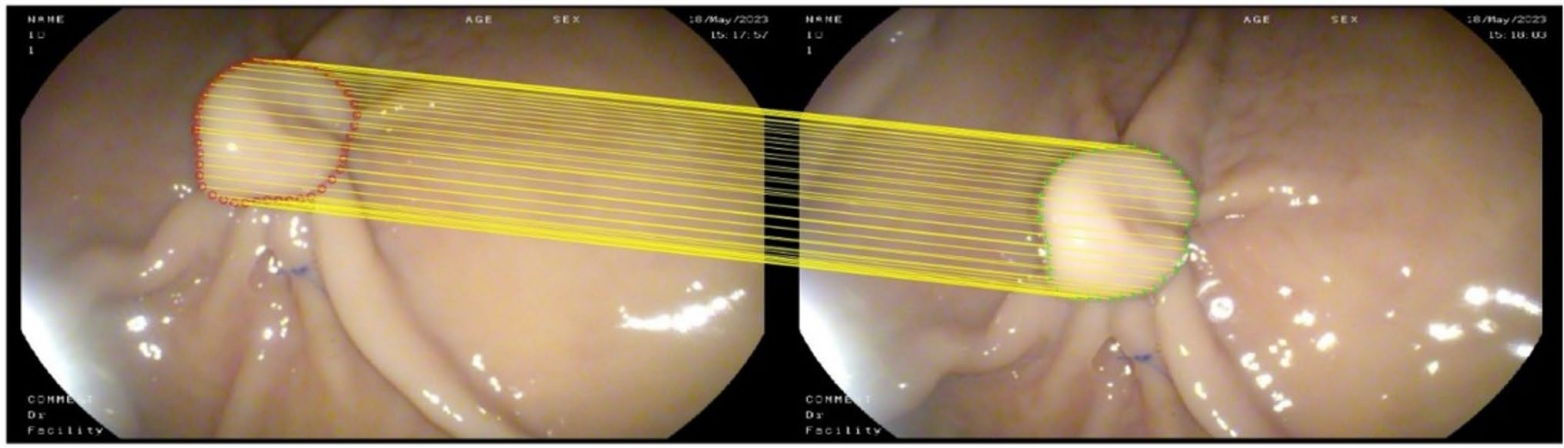
An example of finding corresponding points (down sampled) using shape context on a polyp border in an image pair in ex vivo experiment

Two shapes of the polyp border in two endoscopic images are presented by sample points. Considering a log-polar coordinate system; each shape context is a log-polar histogram of the coordinates of the rest of the point set measured using a reference point as the origin of the log-polar coordinate system. As shown in Fig. 3, the shape context of relatively similar points (i.e. green and red) on the polyp border is visually more similar, while the shape context of another point (i.e. blue) is quite different. This concept was used to find corresponding points along the border of a polyp in the present study. The result of finding corresponding points for one polyp in the pig stomach experiment using this approach is illustrated in Fig. 4.

### Experimental design

The sample size was determined based on the correlation coefficient and considered a two-tailed hypothesis: Null Hypothesis: The correlation coefficient is zero. Assuming a power of 0.8 (β = 0.2), a significance level of α = 0.05, and an anticipated correlation coefficient of C = 0.7, a preliminary sample size was calculated. To account for unforeseen circumstances, an additional 10% was added to this initial calculation, resulting in a final sample size of 15.

**Fig. 5:**
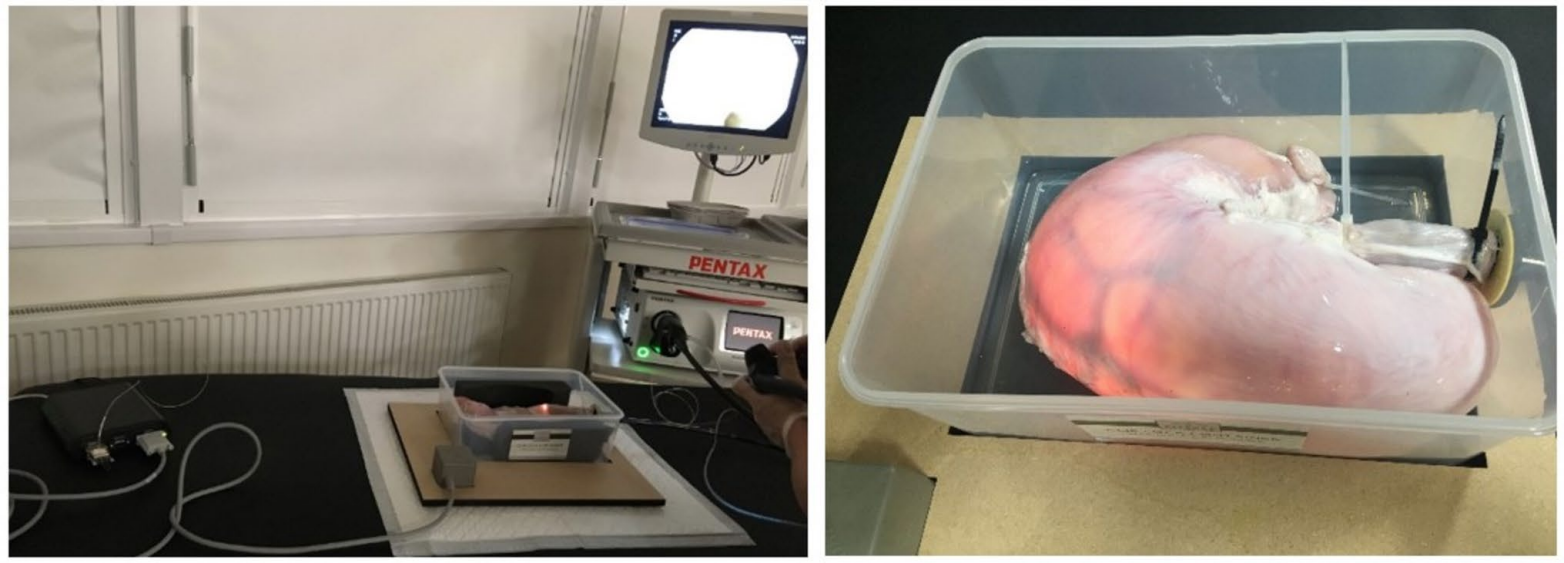
Demonstration of the set up for performing ex vivo pig stomach experiment

Five pig stomachs were utilised in one of which three polyps were created by tying off sections of the stomach wall, in various sizes representing the common real polyp sizes. For this validation, endoscopy procedure was simulated on ex vivo pig stomachs, which were placed in a container and secured at the oesophagus end and sealed at the pylorus end, as depicted in Fig. 5. Notably, the stomach remained unfixed, permitting potential random movements. This procedure, executed by an experienced expert, aimed to mimic real endoscopy in terms of navigation and environment. The stomach was inflated with air and actions such as water jet cleaning and suctioning were performed to replicate actual endoscopic conditions.

The extracted recommendations from model experiment in our previous work were used as a feedback mechanism to achieve the higher accuracy. Five different images of each polyp in a selected perspective made by the expert were taken and used for size and location estimation by the proposed quantitative method. The location of the oesophagus-gastro junction was also recorded, and the locations of the polyps with respect to the junction were determined using the proposed method. This procedure was repeated twice to compute the test–retest reliability for both size and location estimations. After each simulated endoscopy, polyps were measured with a digital calliper. Because the polyps are asymmetric, viewing perspective can affect length measurements. To reduce bias, we aligned the calliper perspective with the captured images. Although calliper measurements are not the most reliable for irregular and non-rigid polyps, we compared them to our method for validation, expecting general agreement between both techniques.

## Result

### Test–retest reliability

The fifteen polyps examined in this study had an average size of approximately 12 ± 4.3 mm (mean ± SD), ranging from 5—20 mm. The Intraclass Correlation Coefficient (ICC) for polyp size estimation between test–retest data was assessed using a two-way mixed effects model [30]. The ICC value for absolute agreement was 0.991 (95% CI 0.973 to 0.997, *p* < 0.05). To explore the agreement of test–retest data for both size and location estimations, we utilised Bland & Altman (B&A) analysis [31]. The differences in the B&A plot can be expressed as units or percentages (i.e. (test–retest)/average %). Given the standard deviation of the differences (s) and the mean of the differences (d), the two upper and lower agreement limits were computed as d ± 2s. Considering the normal distribution of the differences, 95% of differences are expected to lie between d + 1.96s and d − 1.96s.

The normal distribution of differences for test–retest data was confirmed using the Shapiro–Wilk test [32]. Assuming a two-tailed test with a *p*-value of 0.05, the confidence intervals for the mean line were derived.

**Fig. 6:**
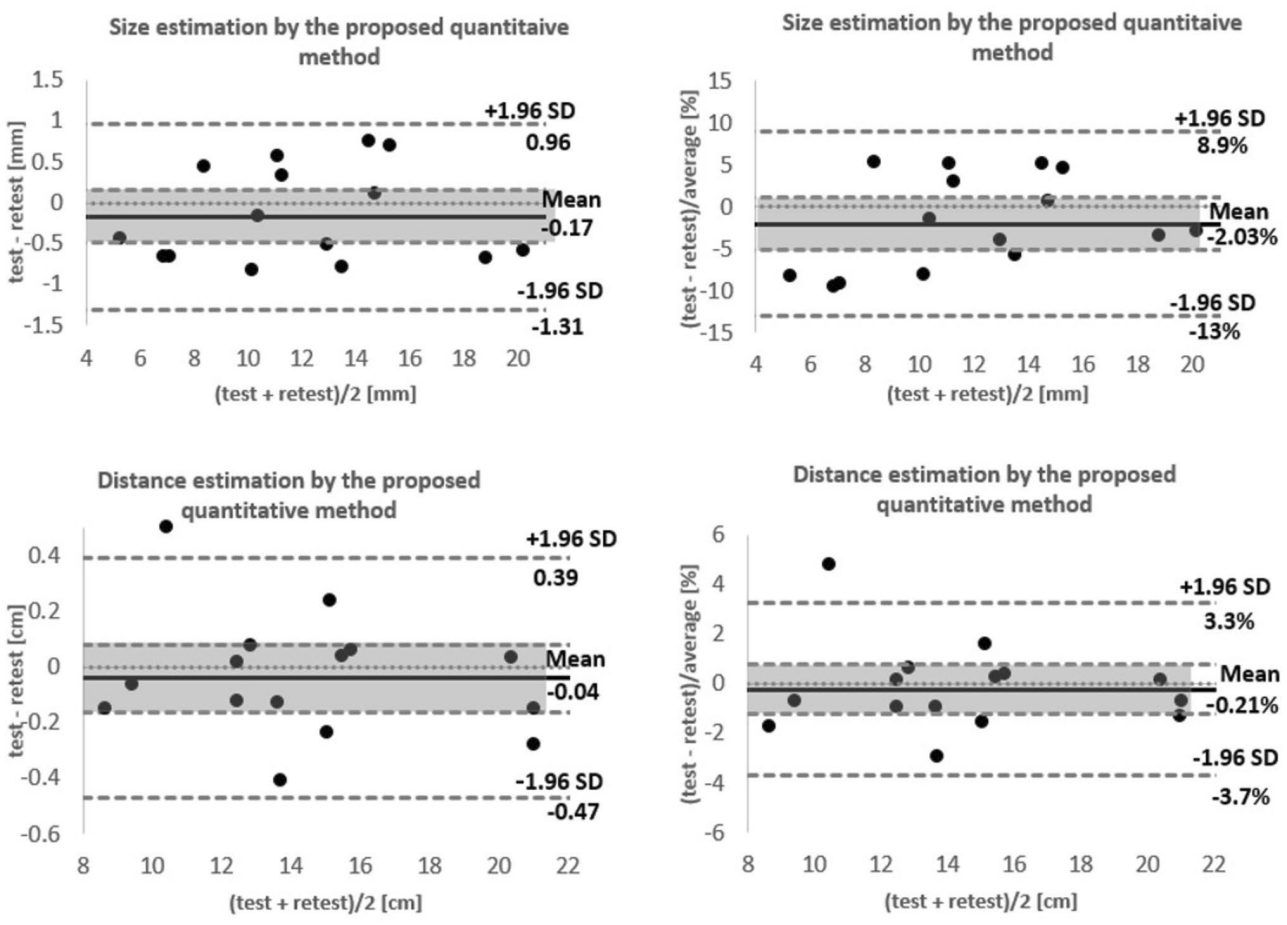
Bland and Altman plot comparing test–retest measurements by the proposed quantitative method. First row: polyp size estimations where differences are presented as units (left) and where differences are presented as percentages (right). Second row: polyp location estimations where differences are presented as units (left) and where differences are presented as percentages (right)

Figure 6 shows the Bland & Altman plots for both size and location estimations using the proposed method, presented in both unit and percentage forms. For size estimation, the mean difference stands at − 0.17 mm or − 2.03%. This difference is not statistically significant, given that the line of equality lies within the 95% confidence interval. Consequently, 95% of the differences between test–retest data lie between − 1.31 and 0.96 mm or from − 13 to 8.9%.

For the estimation of polyp location (i.e. the distance from the oesophagus-gastro junction), the Bland & Altman plot indicates a mean difference of − 0.4 mm or − 0.21% between test–retest data, which is not statistically significant. The limits of agreement, considering a 95% confidence interval, range from − 4.7 to 3.9 mm or from − 3.7 to 3.3%.

### Comparison with clipper measurements

**Fig. 7:**
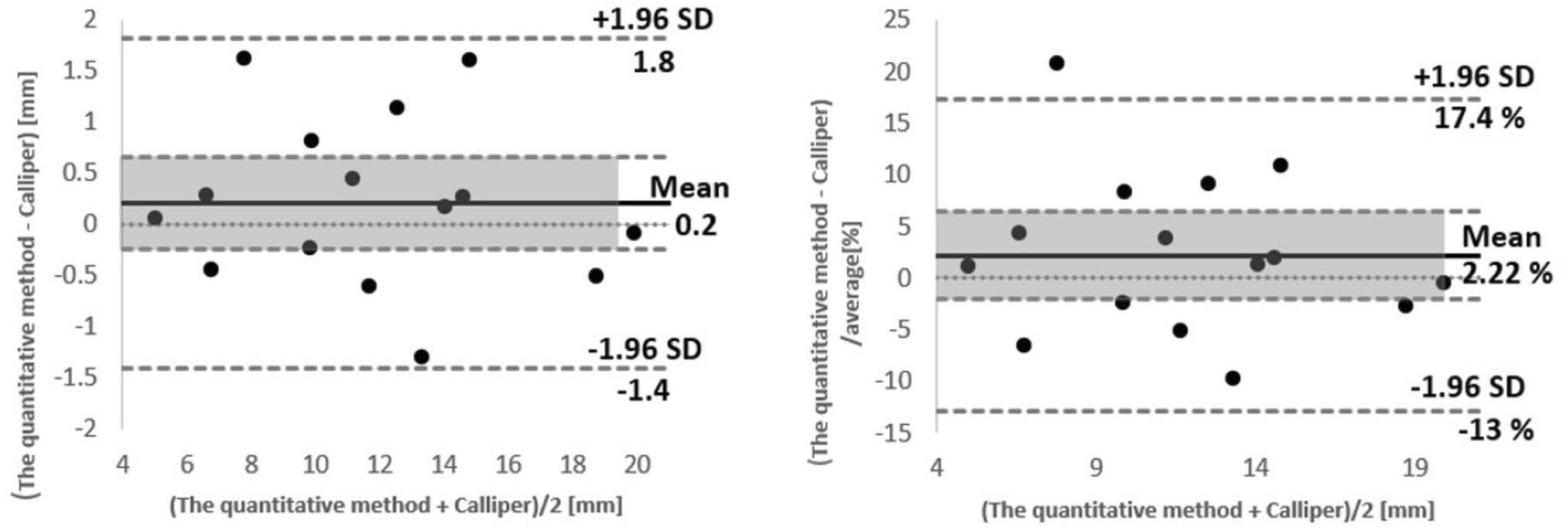
Bland and Altman plot comparing two measurements for polyp size estimation by the proposed quantitative method and digital Calliper where differences are presented as units (left) and where differences are presented as percentages (right)

Figure 7 presents the Bland & Altman plot comparing polyp size estimations made by the proposed method and the calliper. According to the plot, no significant bias is evident (0.2 mm or 2.22%), with 95% of differences fall between − 1.4 and 1.8 mm, or − 13 to 17.4%.

Additionally, a comparison of polyp estimations from the proposed method with those from digital calliper measurements revealed a root mean square difference (RMSD) of 0.81 mm. A median absolute difference of 0.45 mm with an interquartile range (IQR) of 0.88 mm was observed. This difference was not statistically significant (*p* = 0.37). The mean absolute percentage of difference was recorded as 6 ± 5.8%.

## Discussion

In this study, we validated a method developed in our previous work for determining the size and location of polypoidal lesions using a monocular endoscope combined with an electromagnetic tracking sensor through an ex vivo experiment. The test–retest results for size estimation yielded an ICC of 0.99 (*p* < 0.05). A non-significant mean difference of − 0.17 mm, with 95% of differences falling within the range of − 1.31 to 0.96 mm, indicates high reliability. This represents a significant enhancement in comparison to the inter-physician variability seen with visual estimation, which can deviate by more than 5 mm or yield an ICC as low as 0.13 [10].

Moreover, our proposed method demonstrated high reliability in location estimation, with a non-significant bias of − 0.4 mm and 95% agreement limits ranging from − 4.7 to 3.9 mm. In a recent study [8], visual estimations by endoscopists were compared to post-resection before fixation measurements of the polyp, considered as actual value. Based on their Bland & Altman analysis, an agreement limit of − 2.5 to 2.8 mm was reported that was more accurate than pathologic report. When comparing our method with calliper measurements, an agreement range of − 1.4 to 1.8 mm was observed. This underscores that polyp size estimation, using our proposed method, can more accurately represent polyp size, offering an improvement compared to both visual estimations and pathological reports.

In comparison to other device-based methods, Table 1 encapsulates various recent studies focussed on device-based approaches for polyp size measurement. These studies are summarised in terms of their method, accuracy, subjectivity, and estimation time.

**Table 1:** Comparison of device-based methods for endoscopic polyp size measurement

Study	Measuring device & method	Accuracy	Subjectivity	Measuring time
[22]	Addition of an optical probe to an endoscope. Depth estimated based on reflected light and virtual grid adjustment	Mean absolute percentage error of around 9%	Comparison of polyp size with a projected grid	Comparable to visual estimation
[20, 21]	Integration of a laser emitter to an endoscope, projecting a virtual scale on the polyp. Depth estimated based on laser point position and scale adjustment	Mean absolute percentage error of 5.3 ± 5.5%	Comparison of polyp size with a virtual scale	Longer than visual estimation
[18, 19]	Integration of a pattern projector to an endoscope. Real-scale 3D reconstruction based on deformation of a known projected pattern and knowledge of the projector’s location relative to the endoscope camera	Median estimation for absolute error of 1.5 mm with an IQR of 1.67 mm	Manual determination of a line on the image corresponding to the longest length of polyp	Longer than visual estimation
Ours	Integration of an electromagnetic sensor to an endoscope	Median estimation for absolute error of 0.45 mm with an IQR of 0.88 mm Mean absolute percentage error of 6 ± 5.8%	–	Comparable to visual estimation

Acknowledging the varied evaluation settings across studies, our proposed method demonstrates better accuracy in endoscopic polyp size measurement. The method is advantageous due to its minimal impact on the endoscopy procedure as it involves only taking pictures of the polyp, which is a step already recommended in clinical guidelines. Nonetheless, it needs to be further validated by testing in human subjects during endoscopic procedures. Measurements can be estimated immediately after acquiring the endoscopic images of the polyps, aligning with the time required for visual estimation. The proposed method identifies the polyp border and corresponding points automatically, enhancing the level of objectivity as the estimations do not involve comparisons with a scale. Moreover, our method also explored the potential of polyp localisation showed good reliability without necessitating modifications to the existing equipment or procedure.

While our approach demonstrates promising results in the ex vivo environment, the findings of the current study are limited by the fact that a clinical trial on human subjects has not been carried out. It is important to note that the current setup, with the sensor attached externally, does not reflect the final intended design for clinical application. In the real-world application, the sensor will be fully incorporated in the endoscope’s structure internally. Considering the sensor’s small size (1.8 mm in diameter), we anticipate there will be minimal increase in the endoscope’s diameter, post-integration, to allow the endoscope to be used normally describing a non-regular shape using a single-length measurement can introduce variability into the assessment. Using an alternative metric, such as volume, to evaluate asymmetric morphology may potentially reduce both inter and intra rater variability. Even when a method provides the ability to reconstruct the shape, the longest length is usually extracted from that shape because clinical guidelines primarily emphasise the longest length of polyps. To the best of the author’s knowledge, clinical guidelines do not establish a clinical connection or cut-off for lesion volume in relation to clinical decisions.

Additionally, it is worth noting that shape reconstruction comes with certain challenges, including longer computation times and challenges when there are texture-less surface and restricted endoscope movement, which affect the shape reconstruction approach more than the chosen approach in this study. Nonetheless, it is important to highlight that the proposed quantitative method has the potential to be further improved in future research for full 3D reconstruction of the endoscopic view, shape reconstruction, and volume measurement.

Furthermore, the applicability of the proposed quantitative method extends beyond its use in this study; it can be easily generalised to other endoscopic imaging modalities where assessing the size and location of abnormalities is crucial for evaluation and treatment planning, for example, to measure the length of Barrett’s oesophagus. Additionally, the integration of this measurement system with an AI-based diagnosis system could potentially equip clinicians with more comprehensive analysis. Combining size measurement with other morphological and textural features with an AI-based algorithm could improve the risk stratification of polyps. This can help in reducing unnecessary biopsies or polypectomies for polyps that are identified to be benign. It will also help avoid missing potentially malignant polyps as a result of under-estimation of its size. The optimisation of endoscopic procedures will consequently benefit the patient as well as the healthcare system.

The integration of this technology undoubtedly increases the initial cost of the endoscopic device. However, this should be weighed against its potential to facilitate optimal clinical decision-making. By enhancing diagnostic accuracy and treatment precision, it is also plausible that the use of this technology could lead to a reduction in overall healthcare costs, such as those associated with suboptimal clinical decision-making. Further studies will be recommended to accurately quantify these potential cost savings.

In conclusion, this study validates a novel method for providing polyp size and location measurements during simulated endoscopy in an ex vivo setting. The results of the current study suggest that this approach has substantial promise in improving the clinical accuracy of polyp assessment. This research serves as a significant proof of concept study, paving the way for future clinical investigations and marking a significant advancement in developing endoscopic devices with quantitative capabilities.

## Footnotes

1 https://polhemus.com/applications/electromagnetics/.
